# Apigenin Induced Apoptosis by Downregulating Sulfiredoxin Expression in Cutaneous Squamous Cell Carcinoma

**DOI:** 10.1155/2022/8172866

**Published:** 2022-08-04

**Authors:** Wenhua Wang, Xin Liu, Zhibao Zhang, Mingzhu Yin, Xiang Chen, Shuang Zhao, Lisha Wu

**Affiliations:** ^1^Department of Dermatology, Xiangya Hospital, Central South University, 87 Xiangya Road, Changsha, 410008 Hunan, China; ^2^Hunan Key Laboratory of Skin Cancer and Psoriasis, Xiangya Hospital, Central South University, 87 Xiangya Road, Changsha, 410008 Hunan, China; ^3^Department of Critical Care Medicine, The Third Xiangya Hospital, Central South University, Changsha, Hunan 410013, China; ^4^National Clinical Research Center for Geriatric Disorders, Xiangya Hospital, Central South University, 87 Xiangya Road, Changsha, 410008 Hunan, China; ^5^Institute of Medical Sciences, Xiangya Hospital, Central South University, 87 Xiangya Road, Changsha, 410008 Hunan, China

## Abstract

Cutaneous squamous cell carcinoma (cSCC) is the second carcinoma in nonmelanoma skin cancer (NMSC). Sulfiredoxin (Srx) is an antioxidant protein with a role in maintaining redox homeostasis. And Srx has an oncogenic role in skin tumorigenesis. In the current study, we found that apigenin, as a natural flavonoid, downregulated the expression of Srx protein in cSCC cell lines. Apigenin also inhibited the ability of cell proliferation and migration and induced apoptosis in cSCC cell lines. Our results also showed that apigenin induced apoptosis via the activation of the mitogen-activated protein kinase (MAPK) signaling pathway, as well as downregulated Srx expression in cSCC cell lines. Importantly, the effect of downregulation Srx by apigenin has been rescued with the inhibitor of the MAPK signaling pathway intervention. And induced apoptosis by apigenin was partially attenuated by the addition of MAPK inhibitor, Binimetinib. Our research revealed that apigenin induced apoptosis by downregulation of Srx expression through regulating the MAPK signaling pathway in cSCC cells, thus providing evidence of its applicability as a potentially effective therapeutic agent for cSCC treatment.

## 1. Introduction

Cutaneous squamous cell carcinoma (cSCC) is one of the most common skin tumors, and the number continues to increase [1, 2]. The main extrinsic cause of cSCC is solar ultraviolet radiation, including UVA and UVB [3]. A high UV dose can significantly increase the risk of SCC [4]. Up to 16% of cSCC caused by chronic UV or preexisting actinic keratosis can be metastatic [5]. UV produces oxidative free radicals, and free radicals attack vital biomacromolecules such as proteins, lipids, and nucleic acid and destruct their structures and functions, thus promoting the occurrence of tumors [6]. Therefore, the drug that can improve this kind of pathway is a potential therapeutic strategy.

Apigenin is a familiar antioxidant flavonoid compound, which is extracted from various edible things, such as fruits, vegetables, and Chinese medicinal herbs [7]. Studies have shown that apigenin has antitumor activity in a broad range of tumors [8]. It plays an antitumor role mainly by inducing cell apoptosis, leading to cell cycle arrest, and suppressing cell migration and invasion [9, 10]. However, the antitumor activity of apigenin is rarely reported in cutaneous squamous cell carcinoma (cSCC).

Redox homeostasis is the organism's response to maintain physiological function under different stress states [11]. However, in the tumor microenvironment, the redox state is often unbalanced [12]. Based on our previous studies, Sulfiredoxin (Srx) is one of the most essential antioxidant enzymes endogenously, which is the unique enzyme that decreases the hyperoxidized inactive form of peroxiredoxins (Prxs) [13]. Studies indicated that Srx was overexpressed in a variety of malignant tumors, such as melanoma and prostate cancer [14, 15]. Meanwhile, the expression level of Srx was negatively correlated with the tumor prognosis [16]. We found that Srx was expressed increasingly in DMBA/TPA-induced cSCC. And cell apoptosis was increased in Srx^−/−^ mice compared with wild-type mice in cSCC [17]. A recent study showed that upregulated Srx promoted the stemness and survival of cancer stem cells (CSC), which are the most important part of tumor formation [18].

In the current study, we showed that apigenin induced cell apoptosis in cSCC cells, through regulating the MAPK signaling pathway, and decreased the expression of Srx. Our findings suggest that suppressing the expression of Srx via apigenin might be a potential therapeutic target for cSCC.

## 2. Materials and Methods

### 2.1. Materials and Cell Culture

Apigenin, phorbol 12-myristate 13-acetate (PMA; 12-O-tetradecanoylphorbol-13-acetate, TPA), Binimetinib, SB203580, and dimethyl sulfoxide (DMSO) were purchased from Selleck Chemicals LLC (Selleck). GAPDH antibody was purchased from Proteintech. Actin was purchased from Santa Cruz Biotechnology (Santa Cruz, CA). Phospho-Erk1/2, p44/42 MAPK, p38 MAPK, Phospho-p38 MAPK (Thr180/Tyr182), SAPK/JNK, Phospho-SAPK/JNK (Thr183/Thr185), pro-caspase 3, cleaved caspase 3, and cleaved caspase 8 antibody were purchased from Cell Signaling Technology (Beverly, MA).

The mouse epithelial-derived JB6 Cl 41-5a cell was purchased from the American Type Culture Collection (ATCC) and stored in our laboratory [19]. We cultured mouse skin epithelial JB6 cells with minimum essential medium with L-glutamine (2 mM), 1% penicillin-streptomycin, and 5% fetal bovine serum (BI). For malignant transformation, the epithelial-derived JB6 cells required stimulation with TPA. In the current study, we always added 20 nM TPA in the culture medium for maintaining JB6 cells' malignant phenotype while cells were treated with 0.1% (*v*/*v*) DMSO or apigenin (40 *μ*M or 80 *μ*M) for different time points (6-48 h) at 37°C in a 5% CO_2_ humidified incubator. The human cutaneous squamous carcinoma cell line A431 was cultured in DMEM (BI) supplemented with 10% fetal bovine serum (BI) and 1% penicillin-streptomycin at 37°C in a 5% CO_2_ humidified incubator. A431 cells were also treated with 0.1% (*v*/*v*) DMSO or apigenin (80 *μ*M) for various time points (6-48 h). JB6 and A431 cells were subjected to pretreatment with 5 or 10 nM Binimetinib (MEK1/2 inhibitor) for 1 h and then incubated with 80 *μ*M apigenin together for 8 h. Subsequently, the cell lysate was collected for detecting MAPK signaling pathway protein via western blotting. To analyze cell apoptosis, JB6 and A431 cells were treated with 5 or 10 nM Binimetinib for 24 h and harvested the cell for real-time PCR, flow cytometry assay, and apoptotic proteins by western blotting.

### 2.2. Western Blotting

Cells were harvested with Cell Scraper and lysed in RIPA Lysis Buffer (medium) (DingGuo) with a protease inhibitor and phosphatase inhibitors (Selleck), and protein concentration was quantified (2-3 *μ*g/*μ*L) using a BCA assay kit (Beyotime). Protein samples (30 *μ*g) were loaded in each lane of an 8%-12% SDS-polyacrylamide gel (SDS-PAGE) and then transferred onto polyvinylidene fluoride membranes (Millipore). The membranes were blocked with 5% nonfat milk for one hour at room temperature and then incubated with the following primary and secondary antibodies. The blots were detected and analyzed using a gel image analysis system (LI-COR). The membranes were also probed with housekeep proteins (*β*-actin or GAPDH) to normalize the differences between the samples. The intensity of western blotting bands was quantified with the software ImageJ.

### 2.3. Real-Time PCR

Total RNA was extracted from cells using TRIzol reagent (Bioteke Corporation), then reverse transcribed into cDNA using HiScript II Q RT SuperMix for qPCR (Vazyme) according to the manufacturer's instructions. Then, 40 cycles of quantitative reverse-transcription PCR (qRT-PCR) were developed in 96-well plates using SYBR Green qPCR Mixture (CWBIO) on the QuantStudio3 Real-Time PCR System. The fold change of gene expression was calculated by 2^−(ΔCt experimental group–ΔCt control group)^. The experiment was conducted three times independently. We used the sequence of primers including Srx (mouse): forward: 5′-CCCAGGGTGGCGACTACTA-3′, reverse: 5′-GTGGACCTCACGAGCTTGG-3′; Srx (human): forward: 5′-CAGGGAGGTGACTACTTCTACTC-3′, reverse: 5′-CAGGTACACCCTTAGGTCTGA-3′.

### 2.4. Immunofluorescence Assay

Cells (1 × 10^5^/well) were seeded on coverslips in 6-well plates and cultured overnight. After sticking, cells were treated with a combination of apigenin (80 *μ*M) and TPA (20 nM) for 6, 12, 24, or 48 hours, respectively. Cells were washed with PBS, fixed in 4% paraformaldehyde on ice for 15 minutes, and infiltrated with 0.5% Triton X-100 for 5 minutes. After blocking with 5% bovine serum albumin (BSA) for 1 hour, cells were incubated with SRX1 (1 : 100, Proteintech) overnight at 4°C and secondary antibody Alexa Fluor 488 donkey Anti-rabbit (1 : 1000, Invitrogen) and then stained with DAPI (1 : 10, Servicebio) to visualize nuclear DNA. The images were captured by a confocal laser scanning microscope (TCS-SP8; Leica Microsystems) and analyzed.

### 2.5. Cell Counting Kit-8 Assay

Cells (2 × 10^3^/well) were seeded in a 96-well plate and arranged to adhere overnight at 37°C in a 5% CO_2_ humidified incubator. Then, the cells were treated with a combination of TPA (20 nM) and various concentrations of apigenin (from 20 to 120 *μ*M) for 0-96 hours. The effect of a combination of TPA and apigenin on cell viability was tested using the Cell Counting Kit-8 (Bimake) according to the manufacturer's instructions.

### 2.6. Colony Formation Assay

Cells were seeded into 6-well plates (1 × 10^3^ cells/well) and incubated overnight. Then, the cells were allowed to expose to a combination of apigenin (80 *μ*M) and TPA (20 nM) for different times (6 h-48 h). Then, the drug-containing medium was removed and added complete growth medium instead for two weeks until the visible colony formation. During the two weeks, the medium was refreshed every three days. Finally, the cells were washed with PBS, fixed with 4% paraformaldehyde (Servicebio), and stained with 0.5% crystal violet (DingGuo). Then, we took the pictures and analyzed them.

### 2.7. Wound Healing Assay

Cells were seeded into 6-well plates (4 × 10^4^ cells/well for JB6 cell and 5 × 10^4^ cells/well for A431 cell) and cultured overnight. Until cell density was up to 80%, wounds were created by scratching the confluent cell monolayer using a 10 *μ*L plastic pipette tip, and any loose cellular debris or detached cells were removed by washing with PBS. The cells were incubated with or without apigenin (80 *μ*M) or TPA (20 nM), which were both diluted with DMEM containing 2% fetal bovine serum. After being cultured for 6, 12, and 24 hours, the gaps in the wounds were observed with optical microscopy and digitally photographed under 100x magnifications. Each experiment was performed in triplicate. The figures were analyzed quantitatively by ImageJ.

### 2.8. Cell Apoptosis Assay and Flow Cytometry

Cell apoptosis was detected by flow cytometry. Cells were treated in the same way as previously described. The treated cells were digested by trypsin solution without EDTA (Beyotime Biotechnology), washed with PBS, and stained with a combination of 3 *μ*L annexin V and 5 *μ*L propidium iodide (BestBio, Annexin V-FITC Apoptosis Detection Kit) on ice for 15 min before being detected. Resuspended cells were stained by CD24 (Invitrogen, MA511828) and APC-CD44 (BioLegend, 103012) for 30 min at 4°C. The samples were run on a DxP cytometer (Cytek), and the data were analyzed by FlowJo 10.

### 2.9. Statistical Analysis

All statistical analyses were performed with GraphPad Prism6.0 software. Dual comparisons were made with the two-tailed Student unpaired *t*-test. And one-way analysis of variance (ANOVA) with the Newman-Keuls post hoc test was used for multiple comparisons of the means of quantitative data. All experiments were repeated at least three times. The data represent the mean ± SEM. A *p* value of <0.05 (two-tailed) was considered statistically significant for all tests.

## 3. Result

### 3.1. Apigenin Can Downregulate Srx Expression in cSCC Cells

**Figure 1:**
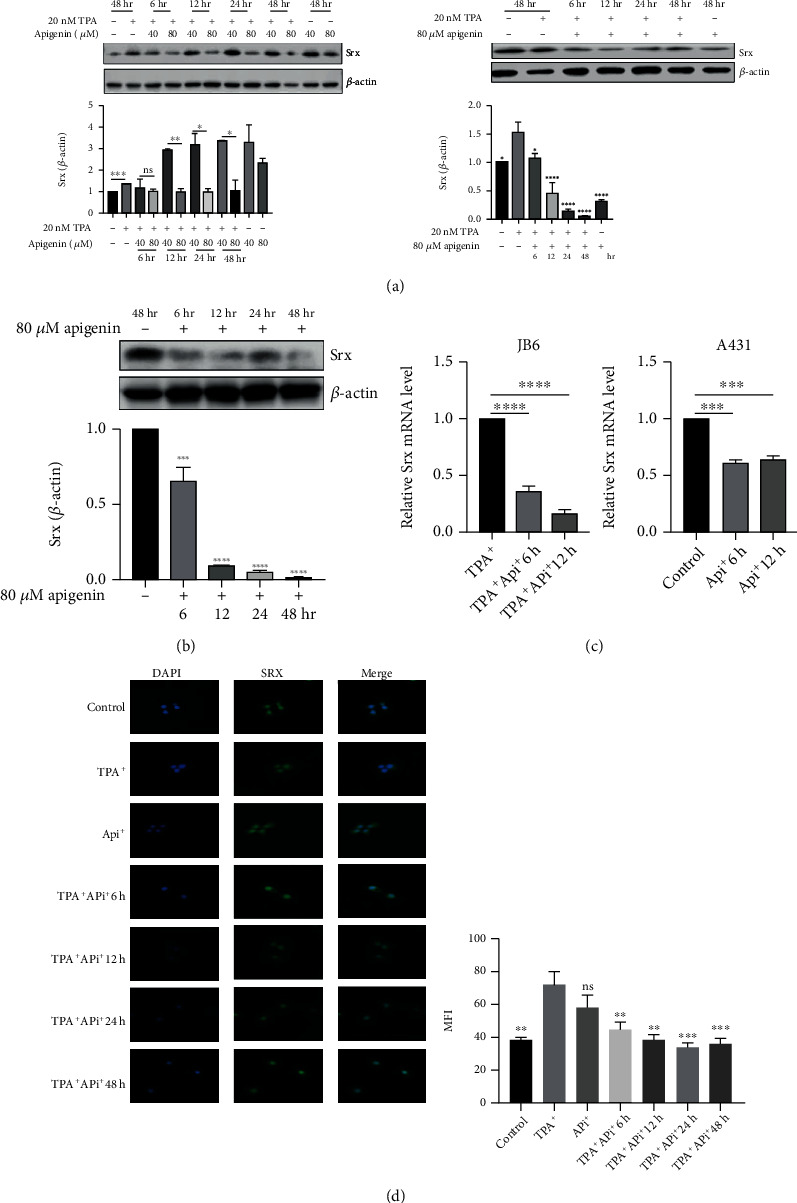
Apigenin can downregulate Srx expression in TPA-induced JB6 and A431 cSCC cells. (a) JB6 was treated with control (DMSO), TPA (20 nM), or apigenin (40 or 80 *μ*M) for different time points (6 h-48 h). Srx expression was detected by western blotting (left). Western blot was served to analyze the expression of Srx while TPA-induced JB6 cells were incubated with apigenin (80 *μ*M) for different times (6 h-48 h) (right). (b) Human SCC A431 cells were treated with apigenin at different times, and the expression of Srx was detected by WB. (c) The mRNA level of Srx in TPA-induced JB6 and A431 cells was conducted to measure after incubation with 80 *μ*M apigenin for 6 h and 12 h. (d) Representative images of immunofluorescence staining of Srx in TPA-induced JB6 treated with control (DMSO) or 80 *μ*M apigenin for 6 h-48 h (left). Quantitative analysis of Srx means fluorescence intensity (MFI) (right) (mean values ± SEM, *n* = 3). Significant differences were evaluated using a one-way ANOVA. ^∗∗^*p* < 0.01, ^∗∗∗^*p* < 0.001, and ^∗∗∗∗^*p* < 0.0001*vs.* control. For JB6 cells, TPA-induced sample as control.

Our previous study indicated that loss of Srx protected mice in DMBA/TPA-induced skin tumorigenesis [17]. To determine whether apigenin can inhibit the expression of Srx, we detected the protein level of Srx in cSCC cell lines via incubation with apigenin at various concentrations for different times. As shown in Figure 1(a), Srx was inhibited while treating TPA-induced JB6 cells with 40 *μ*M or 80 *μ*M apigenin as well as different time points (6 h-48 h) via 80 *μ*M apigenin treatment. Human cSCC cell line A431 was also incubated with or without apigenin, and Srx was also blocked in 80 *μ*M apigenin incubation from 6 h to 48 h (Figure 1(b)). Meanwhile, the mRNA level of Srx in TPA-induced JB6 and A431 cells was significantly decreased after being treated with apigenin (Figure 1(c)). Immunocytochemistry analysis indicated that Srx is mainly expressed in the cytoplasm. As shown in Figure 1(d), we found that Srx located the cytosol and nuclear in TPA-induced JB6 cells. And the cytoplasmic intensity of Srx in JB6 cells gradually decreased with incubating apigenin extended (from 6 h to 48 h). These results demonstrated that apigenin inhibited the expression of Srx in cSCC cell lines.

### 3.2. Apigenin Can Attenuate Cell Proliferation and Migration in cSCC Cells

**Figure 2:**
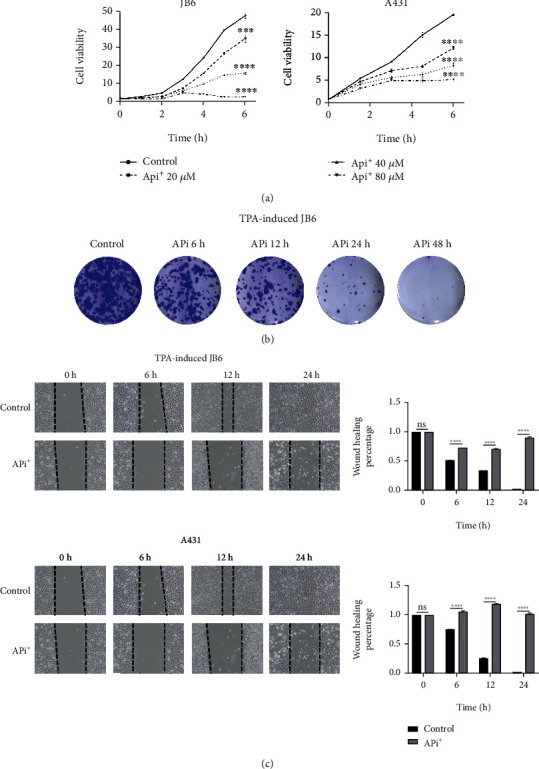
Apigenin can attenuate cell proliferation and migration in cSCC cells. (a) CCK-8 assay was implied to analyze the cell viability after the treatment of TPA-induced JB6 and A431 cells with different concentrations of apigenin (up to 80 *μ*M) as time gone (mean values ± SEM, *n* = 6). ^∗∗∗∗^*p* < 0.0001*vs.* control by ANOVA. (b) Representative images of colony formation assay in TPA-induced JB6 treated with control (DMSO) or 80 *μ*M apigenin. (c) Typical pictures (left) and quantitative analysis (right) of wound healing assay in TPA-induced JB6 (up) and A431 (down) cells. Cells were treated with control (DMSO) or 80 *μ*M apigenin for indicated time points. ns: no statistical significance; ^∗∗∗∗^*p* < 0.0001*vs.* control by Student's unpaired *t*-test.

We performed a CCK-8 assay to determine the influence of apigenin on cSCC proliferation. As shown in Figure 2(a), apigenin can weaken the proliferation of TPA-induced JB6 and A431 cells in a time- and dose-dependent manner. A colony formation assay was developed to further confirm the effect of apigenin in cSCC cell lines. TPA-induced JB6 cells were cultured with or without apigenin for 6-48 hours. When treated with 80 *μ*M apigenin, the formation of cell colonies was inhibited significantly which was more obvious with a prolonged time (Figure 2(b)).

Through the wound healing assay, we also observed the migration ability of cSCC with or without apigenin treatment. The result showed that cell migration was also inhibited by apigenin in a dose-dependent manner in TPA-induced JB6 cells and A431 cells (Figure 2(c)). The results indicated that apigenin had an essentially repressive effect on cell proliferation and migration in cSCC including TPA-induced JB6 and A431 cells.

### 3.3. Apigenin-Induced Apoptosis in cSCC Cells

**Figure 3:**
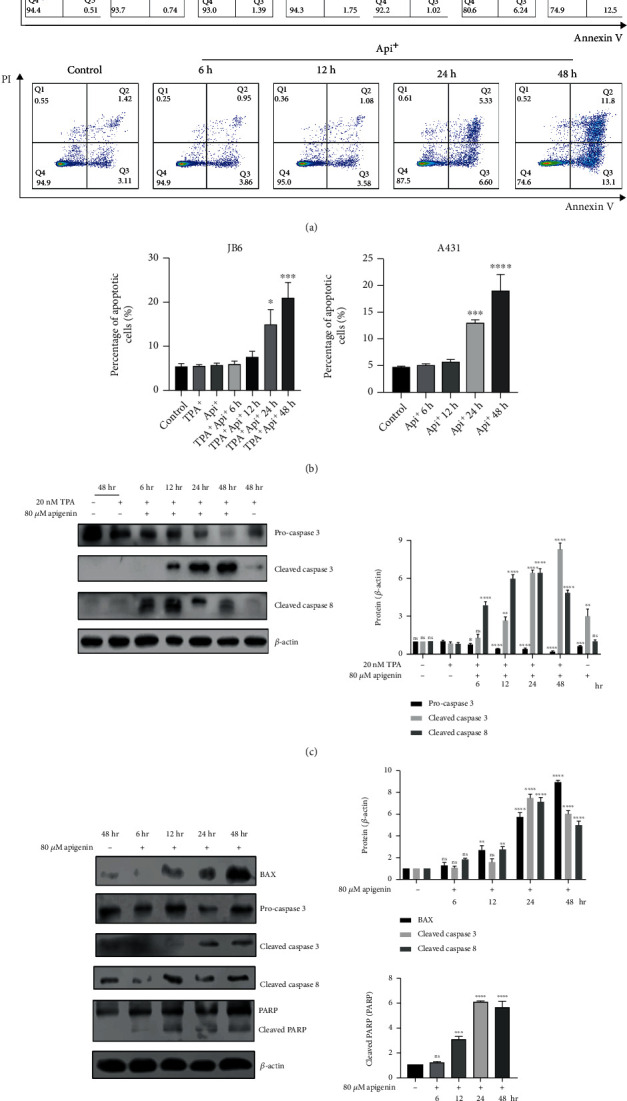
Apigenin induced apoptosis in cSCC cells. (a) Flow cytometry was used to analyze the apoptosis cells. Cells were stained with annexin V and PI to quantify the percentage of apoptotic cells. TPA-induced JB6 cells (upper) or A431 cells (lower) were treated with control (DMSO) or 80 *μ*M apigenin for different times. (b) A concrete percentage of apoptosis cells in TPA-induced JB6 and A431 cells were evaluated using a one-way ANOVA (mean values ± SEM, *n* = 3) ^∗^*p* < 0.05, ^∗∗∗^*p* < 0.001, and ^∗∗∗∗^*p* < 0.0001*vs.* control (TPA-induced sample as control for JB6 cells). (c) TPA-induced JB6 cells were incubated with control (DMSO) or 80 *μ*M apigenin for 6 h-48 h. Western blot was served to analyze the expression of apoptosis-associated proteins (left). The bar graphs on the right showed the intensity of the protein band from each treatment relative to the housekeeping protein (*β*-actin). Valued represent the means ± SEM. Significant difference was designed by ANOVA, ^∗^*p* < 0.05, ^∗∗^*p* < 0.01, ^∗∗∗^*p* < 0.001, and ^∗∗∗∗^*p* < 0.0001*vs.* control (only TPA-induced sample). (d) The apoptosis-associated proteins were detected while 80 *μ*M apigenin was used for treatment for different time-points in A431 cells. The bar graphs showed the intensity quantification of the protein band relative to the housekeeping protein. Significant difference from control by ANOVA, ^∗∗∗^*p* < 0.001 and ^∗∗∗∗^*p* < 0.0001.

To further investigate the mechanism of cell death induced by apigenin in TPA-induced JB6 and A431 cells, we detected cell apoptosis via flow cytometry analysis. We found that the proportion of apoptosis cells increased markedly in a time-dependent manner when TPA-induced JB6 cell was incubated with apigenin. Similarly, cell apoptosis was induced by apigenin treatment in A431 cells (Figures 3(a) and 3(b)). Meanwhile, we detected apoptosis-associated proteins. As western blot analysis showed, the expression level of cleaving form of caspase 3 and caspase 8 was increased in a time-dependent manner as well as pro-caspase 3 expression was decreased in TPA-induced JB6 (Figure 3(c)). As shown in Figure 3(d), proapoptotic proteins including BAX, cleaved caspase 3, cleaved caspase 8, and cleaved PARP were also remarkably increased while A431 cells were incubated with apigenin at different time points. These results indicated that apigenin induced apoptosis in cSCC cell lines including TPA-induced JB6 and A431 cells.

### 3.4. Apigenin-Induced Cell Apoptosis via Regulating the MAPK Signaling Pathway in cSCC *In Vitro*

**Figure 4:**
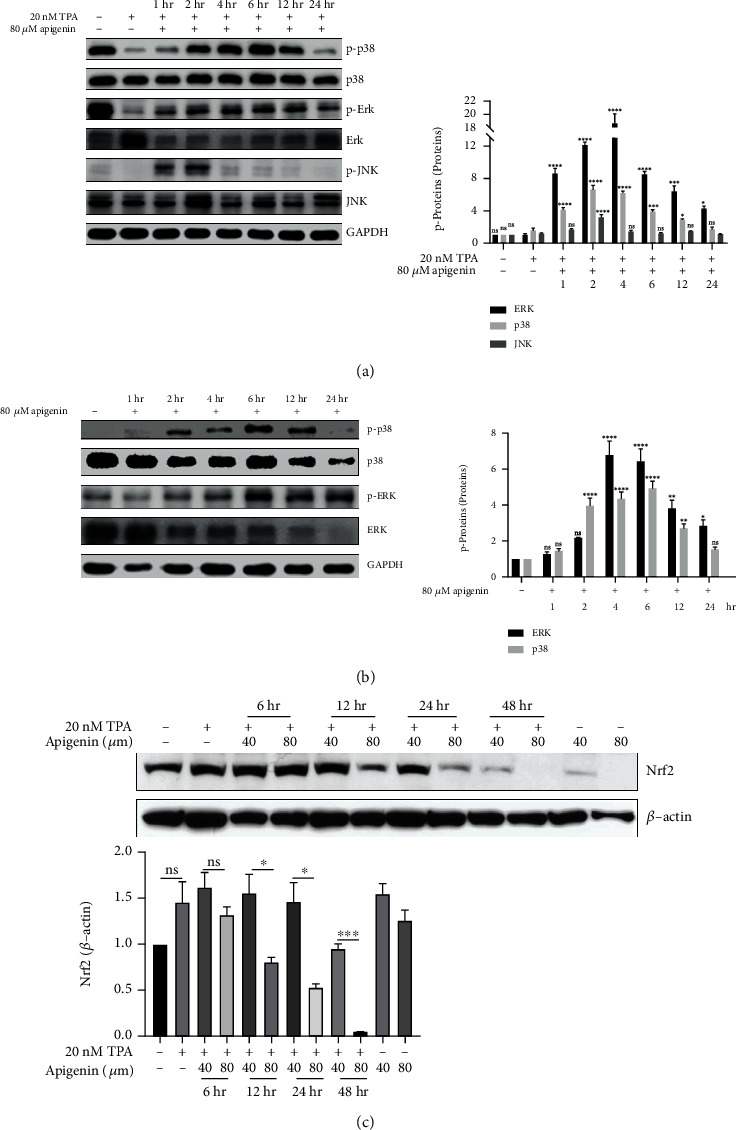
Apigenin activated the MAPK signaling pathway in cSCC in vitro. (a, b) Variation of the MAPK signaling pathway with the treatment of apigenin in TPA-induced JB6 (a) and A431 cells (b). The cells were treated with 80 *μ*M apigenin for different time points up to 24 h. Western blot was applied to analyze the expression of MAPK pathway-associated proteins, includingp38, ERK1/2 and JNK compared with GAPDH (left). The bar graph on the right showed the intensity of the phosphorylation protein band from each treatment relative to the total protein. Valued represent the means ± SEM. Significant difference was designed by ANOVA, ^∗^*p* < 0.05, ^∗∗^*p* < 0.01, ^∗∗∗^*p* < 0.001, and ^∗∗∗∗^*p* < 0.0001*vs.* control (TPA-induced sample as control for JB6 cells). (c) TPA-induced JB6 cell was incubated with control (DMSO, TPA-alone, and apigenin-alone) or apigenin (40 or 80 *μ*M) for indicted time points. Western blot was served to analyze the expression of Nrf2. *β*-Actin was used as the reference for the loading quantity of protein sample. The bar graph indicated the density quantification of the Nrf2 band relative to *β*-actin. ^∗^*p* < 0.05 and ^∗∗∗^*p* < 0.001*vs.* TPA-induced control by ANOVA.

To further clarify the mechanism of apigenin-induced apoptosis, we explored possible related signaling pathways. In our previous study, we found that TPA-induced Srx expression was activated through the activation of mitogen-activated protein kinase (MAPK) partially. We detected essential protein expression related to the MAPK signaling pathway. Western blot analysis revealed that after treatment with apigenin in TPA-induced JB6 (Figure 4(a)), there was a significant increase in protein expression of p-p38 and p-ERK as cultivating prolonged and peaked at 6 h. The level of p-JNK expression peaked for 2 h and gradually decreased until undetectable after 24 h treatment. While apigenin was treated with A431 cells (Figure 4(b)), the expression of p-p38 and p-ERK was also elevated significantly and peaked at 6 h incubation. Afterward, the expression of these proteins was slightly decreased over time, while there were no clear changes in the expression of total p38 and JNK in TPA-induced JB6 cells and A431 cells. However, we found that the total protein of ERK in both TPA-induced JB6 and A431 cells was reduced by apigenin treatment (Figures 4(a) and 4(b)).

Some studies demonstrated that the coactivation of nuclear related factor 2 (Nrf2) was associated with the MAPK signaling pathway. Importantly, the expression of Srx is mediated through the Nrf2-dependent transcriptional activation [20]. We found that the expression of Nrf2 was downregulated in a time- and dose-dependent manner in TPA-induced JB6 cells after the treatment with apigenin (Figure 4(c)). The results suggested that expression of Srx via apigenin-induced decrease might be regulated by Nrf2 in cSCC cells.

**Figure 5:**
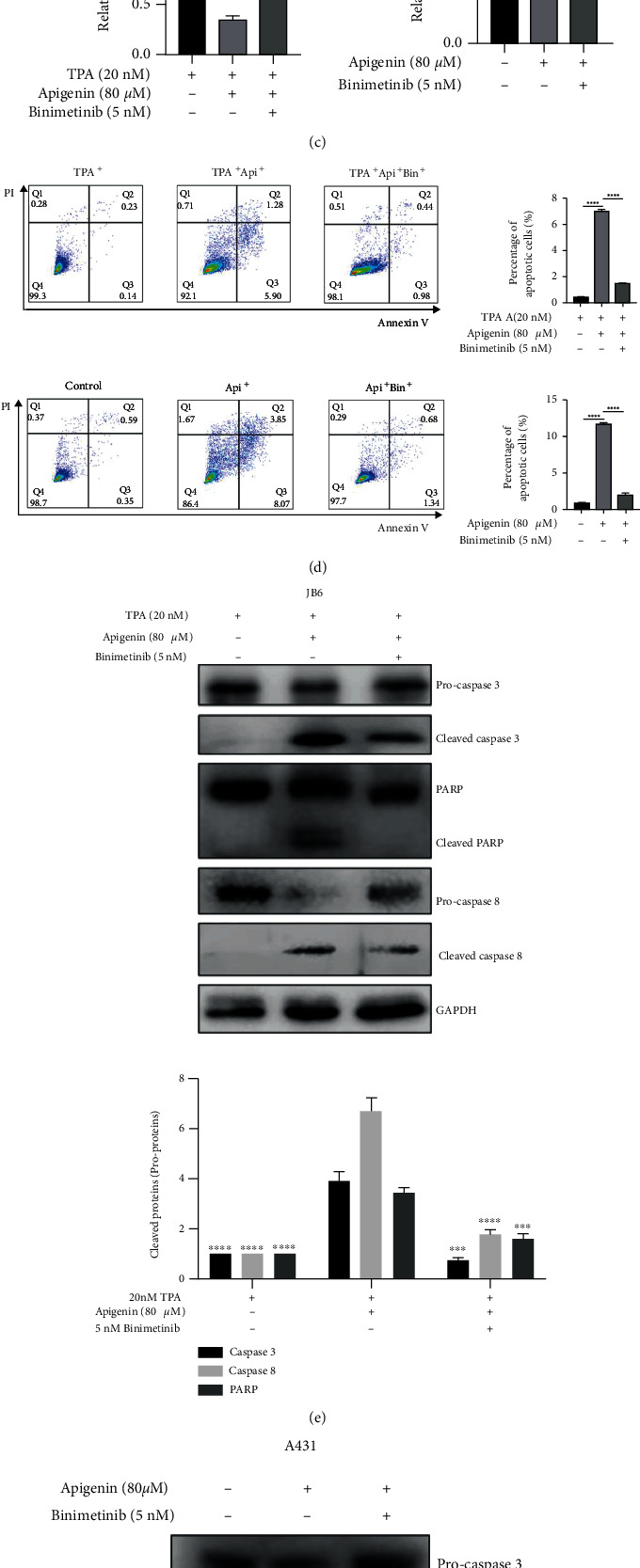
Apigenin induced cell apoptosis and inhibited the expression of Srx via regulating the MAPK signaling pathway in cSCC. (a, b) TPA-induced JB6 cells (a) and A431 cells (b) were treated with the combination of apigenin and inhibitor of MEK1/2, Binimetinib (5 or 10 nM), for 8 h. Western blotting was used to analyze the expression of Srx, p-Erk, and the apoptosis-related proteins BAX and Bcl2. Representative images are shown on the left. The bar graph on the right indicated the intensity quantification of the protein band relative to GAPDH or total protein (ERK). Significant difference was designed by ANOVA, ^∗∗^*p* < 0.01, ^∗∗∗^*p* < 0.001, and ^∗∗∗∗^*p* < 0.0001*vs.* control (TPA-induced sample as control for JB6 cells). (c) Real-time PCR was conducted to quantify the mRNA expression of Srx in cSCC cell lines while cells were treated for indicated compounds. ^∗∗^*p* < 0.01 and ^∗∗∗^*p* < 0.001 vs control by ANOVA analysis. (d) Representative images of cell apoptosis analysis by flow cytometry after 5 nM Binimetinib (MEK1/2 inhibitor) treatment for 24 h, respectively, in TPA-induced JB6 (up) and A431 (down). The percentage of apoptosis cells after apigenin treatment with or without Binimetinib is shown in the bar graph. ^∗∗∗∗^*p* < 0.0001*vs.* control by ANOVA analysis. (e, f) TPA-induced JB6 (e) and A431 (f) were incubated with or without the MEK1/2 inhibitor (5 nM Binimetinib) for 24 h in the presence of 80 *μ*M apigenin. Whole-cell lysates were subjected to western blotting to detect the apoptosis-associated proteins caspase 3, caspase 8, and PARP. The bar graph showed the intensity quantification of the protein bands from each treatment. Significant difference was designed by ANOVA, ^∗∗∗^*p* < 0.001 and ^∗∗∗∗^*p* < 0.0001*vs.* the apigenin-alone group.

To further confirm the influence of the MAPK signaling pathway in apigenin-treated cSCC cell lines, we conducted the rescue assay. We used Binimetinib (MEK1/2 inhibitor) treatment TPA-induced JB6 and A431 cells with apigenin together. First, we found that treatment with Binimetinib restored the inhibiting effect of Srx protein and mRNA expression by apigenin (Figures 5(a)–5(c)). Meanwhile, activation of MAPK during apigenin-induced apoptosis was further confirmed. Binimetinib treatment markedly suppressed proapoptotic protein BAX as well as elevated antiapoptotic protein Bcl2 (Figures 5(a) and 5(b)). Upon flow cytometry (FACS) analysis, Binimetinib treatment dramatically reduced the percentage of apoptosis cells compared to apigenin-alone treatment (Figure 5(d)). Importantly, we also detected the expression of several apoptotic proteins including caspase 3, caspase 8, and PARP. The results showed that the cleaved level of caspase 3, caspase 8, and PARP was downregulated after MEK1/2 inhibitor addition into JB6 and A431 cells (Figures 5(e) and 5(f)). The results implied that after the inhibitor's intervention, cell apoptosis was suppressed in TPA-induced JB6 and A431 cell lines, which demonstrated that cell apoptosis induced by apigenin was partially rescued via MAPK inhibitor addition. Taken together, our results revealed that apigenin-induced Srx downregulation was regulated by the MAPK signaling pathway. And apigenin induces apoptosis by downregulation of Srx partially via regulating the MAPK signaling pathway in cSCC cells.

## 4. Discussion

Sulfiredoxin (Srx) is a vital antioxidant enzyme [21], which was first discovered in yeast. Srx affects its downstream target gene hyperoxidized Prxs, transforming it to active Prxs with the presence of ATP [22]. Srx plays a critical role in tumorigenesis involved in cell proliferation, migration, and metastasis [23]. According to the previous study, Srx plays an oncogenic role in skin tumorigenesis. And targeting Srx can prevent tumor proliferation by using shRNA and Srx^−/−^ mice [17]. Therefore, our group is interested in possible promising compounds that can inhibit Srx expression and have an antitumor effect at the same time.

Apigenin, traditional medicine for many years, possesses multiple pharmacological effects, such as antioxidant, anti-inflammatory, and antitumor [24, 25]. In the process of antitumor, it works in different ways. As reported, apigenin induces apoptosis and autophagy by inhibiting the PI3K/Akt/mTOR pathway in hepatocellular carcinoma cells [26]. It was also reported that apigenin inhibits histamine-induced cervical cancer tumor growth by regulating estrogen receptor expression [27]. It was also found that apigenin suppresses PD-L1 expression in melanoma and host dendritic cells to elicit synergistic therapeutic effects [28].

In the current study, we found that the expression of Srx in TPA-induced JB6 cells *in vitro* increased and Srx was also highly expressed in A431, which is consistent with previous research results. Importantly, the result demonstrated that Srx was prominently decreased with the treatment of apigenin in TPA-induced JB6 cells and A431 cells. In addition, we also found that apigenin significantly inhibited cell proliferation and migration in TPA-induced JB6 and A431 cells. Also, apigenin induced cell apoptosis in cSCC cells.

Previous studies reported that TPA-induced Srx expression was correlative with the MAPK signaling pathway. MAPK, a large family of serine-threonine kinases, forms major cell proliferation signaling pathways from the cell surface to the nucleus [29], including the extracellular-signal-regulated kinases (ERK MAPK), the c-jun N-terminal kinase or stress-activated protein kinases (JNK or SAPK), and MAPK14 (p38 MAPK). And the dysfunctional MAPK pathway plays an important role in the progression of tumors by affecting cell proliferation, migration, apoptosis, and so on [30]. Studies showed that the activation of nuclear related factor 2 (Nrf2) was associated with overexpression of the MAPK pathway [31]. Moreover, Nrf2 is a transcription factor that upregulates the expression of genes that have an antioxidant effects in their promoter, including Srx [20]. Through western blot analysis, we found that there was a significant increase in protein expression of p-Erk, p-p38, and p-JNK in TPA-induced JB6 and A431 cells with culturing with apigenin. Meanwhile, the expression of Nrf2 significantly decreased in a time- and dose-dependent manner in the cSCC cell lines. Then, we speculated that apigenin could activate MAPK signaling and regulate the expression of transcript factor Nrf2, resulting in downregulation of Srx expression in the cSCC. However, the mechanisms of induced apoptosis by Srx regulation have remained to be further researched in the future.

The MAPK signaling pathway is considered pivotal for cell proliferation, differentiation, and cell apoptosis [32]. Apigenin has been stated to modulate the MAPK signaling pathway. It has been shown that apigenin promoted the growth arrest via downregulation of p-ERK1/2, p-AKT, and p-mTOR [33]. In anaplastic thyroid carcinoma cells, the combination of apigenin and TRAIL resulted in a decreased BCL2 and increased ERK1 and ERK2 expression [34]. Instead, the research has demonstrated that apigenin elevated the levels of ERK1/2 and decreased p-p38 kinase levels in prostate cancer cells [35]. Therefore, these findings have manifested that apigenin can induce apoptosis by regulating the MAPK pathway and is a potential therapeutic option for the treatment of cSCC.

Furthermore, Binimetinib as an MEK1/2 inhibitor could impede the effect of apigenin-induced apoptosis, as well as could restore the expression of Srx by apigenin treatment in cSCC cells. So far, Binimetinib in combination with encorafenib (BRAF inhibitor) is approved in several countries for the treatment of advanced BRAF-mutant melanoma [36]. In melanoma cases, it has been reported that apigenin activates the cleaved caspase-3 and PARP expression sites; downregulates Twist1, MMP-2/9, VEGF, p-mTOR, ERK1/2 proteins, and p-AKT; and deactivate FAK/ERK1/2 pathways [37]. Based on our findings, we suggested that the benefit from apigenin inhibition in skin cancer cells might be limited following MAPK pathway inhibitor.

**Figure 6:**
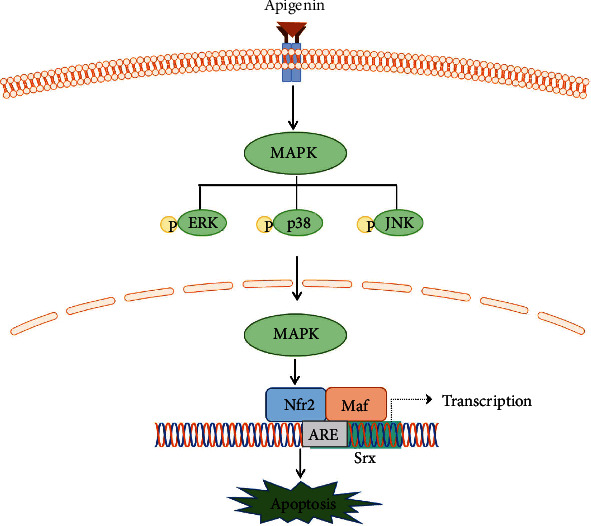
The summarization of the effect of apigenin in cSCC cell lines. After treatment with apigenin, the MAPK signaling pathway was activated gradually through the form of phosphorylation, especially the ERK1/2 pathway. Then, Phospho-MAPK from the cytoplasm to nucleus may generally downregulate the expression of Srx by inhibiting the expression of Nrf2. Then, apigenin might induce cell apoptosis in cSCC cells.

Taken together, our study revealed that apigenin inhibited cell proliferation and migration and induced cell apoptosis via downregulation of Srx and then activation of the MAPK signaling pathway (Figure 6), which indicated that apigenin mediated positive effects in cSCC and supplied a potential therapeutic strategy in the treatment of cSCC patients.

## Data Availability

All data used to support the findings of this study are included within the article.
